# Towards Supply Chain Visibility Using Internet of Things: A Dyadic Analysis Review

**DOI:** 10.3390/s21124158

**Published:** 2021-06-17

**Authors:** Shehzad Ahmed, Tahera Kalsoom, Naeem Ramzan, Zeeshan Pervez, Muhammad Azmat, Bassam Zeb, Masood Ur Rehman

**Affiliations:** 1 School of Business & Creative Industries, University of the West of Scotland, Paisley PA1 2BE, UK; shehzad.ahmed@uws.ac.uk (S.A.); bassam.zeb@uws.ac.uk (B.Z.); 2 School of Computing, Engineering & Physical Sciences, University of the West of Scotland, Paisley PA1 2BE, UK; naeem.ramzan@uws.ac.uk (N.R.); zeeshan.pervez@uws.ac.uk (Z.P.); 3 College of Engineering & Physical Sciences, Department of Engineering Systems and Supply Chain Management, Aston University, Birmingham B4 7ET, UK; m.azmat@aston.ac.uk; 4 James Watt School of Engineering, University of Glasgow, Glasgow G12 8QQ, UK

**Keywords:** IoT, Industry 4.0, supply chain visibility, Supply 4.0

## Abstract

The Internet of Things (IoT) and its benefits and challenges are the most emergent research topics among academics and practitioners. With supply chains (SCs) gaining rapid complexity, having high supply chain visibility (SCV) would help companies ease the processes and reduce complexity by improving inaccuracies. Extant literature has given attention to the organisation’s capability to collect and evaluate information to balance between strategy and goals. The majority of studies focus on investigating IoT’s impact on different areas such as sustainability, organisational structure, lean manufacturing, product development, and strategic management. However, research investigating the relationships and impact of IoT on SCV is minimal. This study closes this gap using a structured literature review to critically analyse existing literature to synthesise the use of IoT applications in SCs to gain visibility, and the SC. We found key IoT technologies that help SCs gain visibility, and seven benefits and three key challenges of these technologies. We also found the concept of Supply 4.0 that grasps the element of Industry 4.0 within the SC context. This paper contributes by combining IoT application synthesis, enablers, and challenges in SCV by highlighting key IoT technologies used in the SCs to gain visibility. Finally, the authors propose an empirical research agenda to address the identified gaps.

## 1. Introduction

The Internet of Things (IoT), considered recent development of information and digital technologies, has provided immense business opportunities in industry, manufacturing, and service provision, restructuring the entire supply chains [1,2]. With the introduction of Industry 4.0 within manufacturing and production environments, the supply chains (SCs) have encountered digitisation and a massive impact in the form of alliance between suppliers, manufacturers, and customers to enable transparency through the product lifecycle [3,4]. This digitisation of SCs, often termed Supply 4.0, enables companies to have an effective and efficient integration between personnel, processes, equipment, and products [5], providing efficiency, flexibility, and visibility [6,7]. Visibility in Supply 4.0, as proposed by Ben-Daya et al. [1], is that it constitutes the capturing of actors transiting the SC in timely instances substantiating their location, identity, and status. Similarly, the observations of Büyüközkan and Göçer [8] and Ellis et al. [9] conceptualise visibility to the degree of information sharing and SC nodes’ access to generate mutual benefits. In a highly visible supply chain (SC), companies will manage their SCs effectively while meeting customer expectations and reducing operational costs [10,11].

Industry 4.0 has opened a new horizon, where technology-oriented manufacturing approaches, computer-integrated manufacturing, lean management, and cellular manufacturing, enabled by the cyber-physical systems (CPSs), have changed the vision of industrial production [12,13,14]. According to Ardito et al. [15], CPSs consist of digital integrations of computers and networks with physical processes, which create intelligent systems that enable the provision of immediate answers to product modifications and failures along the industrial production chain [8,16]. In SCs, Industry 4.0 has become one of the vital factors that can improve industrial performance in the market [17,18]. In Industry 4.0, digitisation promotes more agile, efficient, and customer-focused SCs [14,19].

Supply 4.0 is initiated to concentrate on the connection between Industry 4.0 and SC, and promote the investigation and description of IoT applications in SCs [5,20]. The integration of SCs with IoT and big data (BD) is also known as Supply 4.0. Supply 4.0 is a term coined in which Industry 4.0 has created the digitisation of SCs, enabling them to cope with product requirements changes, making SC operations faster, granular, and precise [21]. Besides achieving flexibility, agility, and resilience, Supply 4.0 has given companies the benefit of significant visibility of the whole SC, a vital momentum to have speedy responses to changes and effective integration between suppliers and customers [1].

The concept of visibility in SCs relates to the ability of a business to track a product from its manufacturing to the consumption stage [11]. Its importance stems from the role of IoT in strengthening SC operations by enabling reliable, consistent, and up-to-date data to all SC stakeholders [22]. Supply 4.0 and IoT also gain prominence in modern business as these technologies enable supplier integration and prioritise stakeholder engagement as the basis for driving supply chain performance improvement through visibility [5]. Using IoT applications, autonomous coordination between things is facilitated, and communication between humans and things in SCs is taken to another level [23]. This is achieved while the objects are stored in a storage facility or transited between different SC bodies [1,24]. IoT has a significant impact on the internal and external integration of the SCs between various processes and with suppliers and customers [25,26]. According to Fawcett et al. [27], supply chain management’s (SCM) key to success is acquiring, exchanging, and elaborating operational knowledge on time. The data produced by smart objects can provide unprecedented visibility into all phases of the SC by effectively collecting, analysing, and turning that data into useful information [9,28,29], providing early indications of internal and external circumstances that need remediation.

Though relatively a newer concept, referring to the evolution of Supply 4.0 and visibility, an underpinning of logistic aspect could be noted. The primary emphasis was on the traceability of the entities involved in the SC [30]. However, the analysis shows that a vivid specification on transparency is lacking. In contrast, the primary concern of the IoT-based aspect of SC visibility is on the organisations’ capabilities to collect and evaluate the information for accomplishing the fit between strategy and goals [31,32]. The interpretations from the inventory and operation perspective highlight how the lack of visibility across both upstream and downstream operations influence the efficacy of the progression of the SC [10,33]. Additionally, a considerable volume of literature on visibility in SCs has entailed the benefits of an organisation’s financial and operational performance affecting inventory, quality, lead time, sales, and costs [30].

A variety of research has been carried out to investigate IoT’s impact on different areas such as sustainability, organisational structure, lean manufacturing, product development, and strategic management [23]. The majority of these studies focus on the contributions and threats of IoT related to flexibility, transparency, information sharing, connectivity, traceability, and tracking within Industry 4.0 [33]. Although comprehensive work has been done in these areas, it has been found that research investigating the relationships and impact of IoT in supply chain visibility (SCV) is minimal.

Moreover, most of the articles on the impact of IoT on SCV remain theoretical. The leading technologies that lead to the improvement of SCV have yet to be identified. No study has tried to summarise the literature on the impact of IoT on SCV, except Haddud et al. [12]; they attempted to identify SCV characteristics using a systematic literature review approach. Nevertheless, that study was confined to only characteristics of SC visibility, so there was a need to systematically explore the links and patterns of previous studies on IoT’s impact on SCV. In addition, although the term Supply 4.0 has grasped the attention of academics, it still lacks in-depth knowledge of the implementation processes for successful transformation. A lack of common ground would help practitioners know which technologies are available to implement Supply 4.0 to gain SCV successfully. In addition, even though the advantages and disadvantages of IoT have been discussed at an academic level, further research is needed in areas of Supply 4.0 to provide a robust and reliable solution for the practical implementation of Supply 4.0 in the context of achieving SCV.

Hence, this paper extends the literature on IoT’s impact on SCV by relying on a systematic literature review. This would include considering the associated variables and functionalities that enhance the value of visibility in SCs. This paper contributes to the concept of SCV by describing the literature highlights in a systematic way that will increase the understanding of IoT and its impact on SCV. Our research provides insights into the literature on IoT and SCV. The results highlight the main themes and concepts in the literature. Besides, some patterns are identified in the literature of IoT and SCV.

This study follows the following approach: first, the steps are adopted to complete a systematic literature review. Next, the research objective and research questions are identified. The research methodology is the following step that consists of selection and evaluation criteria for databases, journals, and articles. After that, qualitative and quantitative analysis and critical findings are given by using various tools. In the last, an overall conclusion is presented.

## 2. Systematic Literature Review

**Figure 1:**
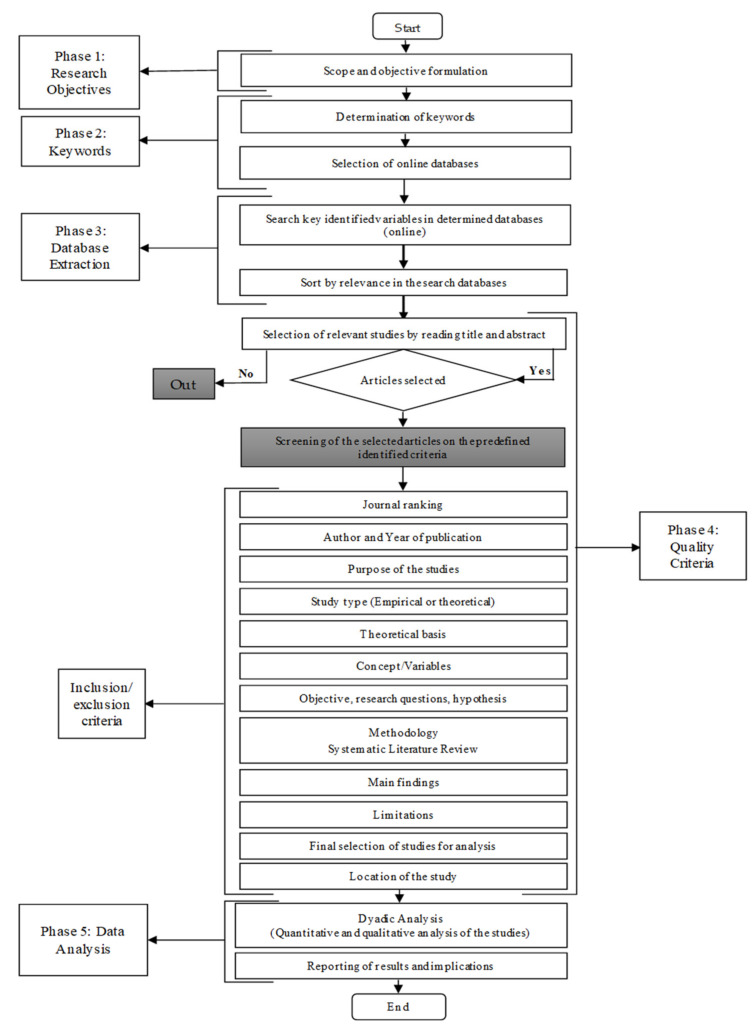
Steps in the systematic literature review (PRISMA flow diagram).

A literature review summarises and examines the existing literature relevant to the topic under investigation [18]. A more rigorous and well-defined scientific approach is used in a systematic review, in contrast to a traditional literature review, to critically analyse the prior literature on a specific research area [34,35]. To conduct a systematic literature review on the impacts of IoT on SCV, an in-depth analysis of existing journal articles was carried out, beginning with determining the number of keywords for online search and online databases identification, following [36] and [37]. The whole selection process is carried out scientifically without the authors’ biases; the overall systematic literature review process is presented in Figure 1 using the Preferred Reporting Items for Systematic reviews and Meta-Analyses (PRISMA) flow diagram. Using a PRISMA flow diagram allows depicting the flow of information through different phases of a systematic review simply and pragmatically [38].

### 2.1. Phase 1: Research Objectives

This study aimed to review the existing literature using a systematic review of IoT’s impact on SCV and identify its drivers and challenges. To achieve the objectives as mentioned above, the following research questions were formed:

1. What role does IoT play in Supply 4.0 and SCV?
2. How does IoT impact SCV?
3. What are the drivers and challenges of adopting IoT within SCs to gain visibility?

### 2.2. Phase 2: Identifying Keywords

During this stage, keywords relevant to the objectives were identified so that this research is appropriately positioned. After multiple brainstorming sessions among the authors, 21 keywords were identified in total (Table 1). Boolean logic was used to enhance validity. The keywords were combined into a series of strings to refine them for the research, such as IoT, IoT technology, visibility, drivers, and challenges. Words such as “IoT AND/OR Supply 4.0”, “Industry 4.0”, “Visibility and IoT”, and “Supply 4.0 AND/OR Visibility” were used. The strings were refined continuously, and 12 relevant search strings were finalised for secondary data search. These keywords and search strings enabled the authors to extract relevant articles which would help meet the research objective.

Moreover, these databases provided high-quality peer-reviewed journal articles with full-length abstracts [36]. After an initial analysis of the databases, it was observed that academics and practitioners alike had shown increased interest in the role of IoT in SCV since 2006; therefore, publication numbers are high during these years [1,3,8,25]. Hence, the period of 13 years (2006–2019) was specified for our research.

### 2.3. Phase 3: Selecting Databases

This phase was concerned with the selection of relevant databases and specifying the period for the publications. In this study, for pursuing a systematic literature review, a search for articles on the importance and use of IoT in achieving SCV was conducted. This search relied on the four central online databases, and these included EmeraldInsight, ScienceDirect, Taylor and Francis, and Web of Science (Table 1). The rationale behind these libraries’ adoption is that they are the most extensive and are more reliable libraries for extracting relevant academic journals [37].

### 2.4. Phase 4: Setting Quality Criteria

Shortlisting of the articles was based on the inclusion criteria, such as assuring quality and reliability; this study relied on a two-stage appraisal process [34].

The first step relied on manual screening. The articles found were arranged by relevance in the online database. After the online search was carried out, the articles were further sorted based on the various criteria mentioned in Figure 1. Furthermore, the studies were sorted by reading abstract, purpose, objectives, hypothesis, methodologies, limitations, and findings to ensure the comprehensive coverage of relevant literature in all aspects of IoT’s impact on SCV.

In the second step, each selected article’s relevance was assessed based on the Association of Business Schools (ABS) ranking of the concerned journal [19]. The articles were selected from the ABS ranked journals. This is because the ABS ranking assures the relevance of the chosen articles. The journals listed in it have already been evaluated based on citation scores, expert judgments, and peer reviews.

The use of this strategy served as a valuable tool for narrowing the search, and as a result, 41 articles were found. Table 2 specified the inclusion/exclusion criteria for this study.

### 2.5. Phase 5: Data Analysis

Data analysis was carried out relying on a novel dyadic method approach, and this involved quantitative systematic literature review (QSLR) and qualitative analysis. The dyadic analysis strategy’s primary benefit is extensive data analysis considering both qualitative and quantitative aspects.

Thus, by using the combination of content analysis and the QSLR, a researcher can infer the different dimensions of the role of IoT in SCV, including new trends, critical success factors, and the challenges associated with the execution of the process. Besides, the use of two approaches helped in juxtaposing the findings. Thus, overall, a researcher can accomplish a nuanced understanding of the research context.

## 3. Quantitative Review

For mapping the quantitative trends in the chosen literature review, this study selected the QSLR. This approach’s adoption was to identify the critical instances’ volume to register their frequency [34]. This novel method of analysis was employed in this research to extract information on the critical methodologies used and generate insights on the distribution of the publication, period, and research focus in the chosen studies. Using vivid steps and using MS Excel, these key quantitative trends across the journals could be highlighted for inferring generalisations on the notion of SCV. Besides, the different factors influencing the adoption of IoT to gain SCV could also be understood.

The evaluation based on QSLR considers the journal’s quantitative dimensions, which provide feedback on the selected material’s descriptive measures. The following criteria were followed, and these included periods, geographic location, methodology, and research theme associated with each selected article. Consideration of these parameters enabled registering the categories and items encoded across the chosen materials.

### 3.1. Time-Period Analysis

**Figure 2:**
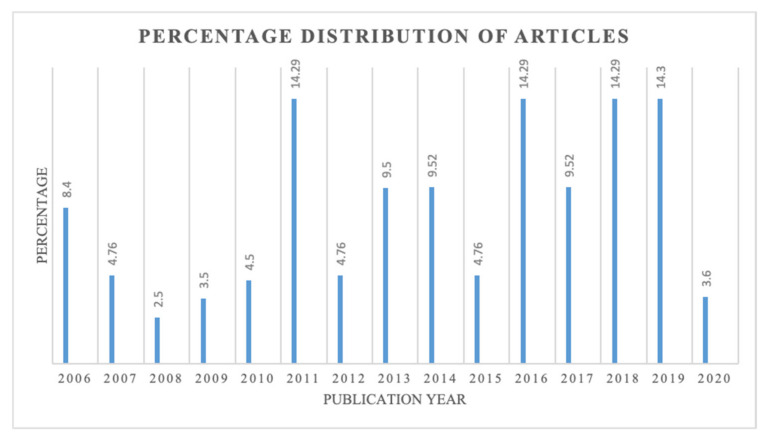
Percentage distribution of articles during 2006–2020.

Considering the period, it could be seen that the distributions of the publications were higher in the periods 2011, 2016, 2018, and 2019. A total of 14.28% of all publications were found in 2011, 2016, and 2018, making up 42.84% of the total (Figure 2). Besides, it has been noted to keep the search in line with the inclusion criteria. One of the significant inferences from the analysis of journals’ distributions during this period is that it reflected a continuity of publications related to the IoT and supply chain visibility research, except in 2008 and 2009. This analysis revealed that research in the domain under investigation has gradually increased since 2006, with the highest publications since 2016.

### 3.2. Geographical Location Analysis

**Figure 3:**
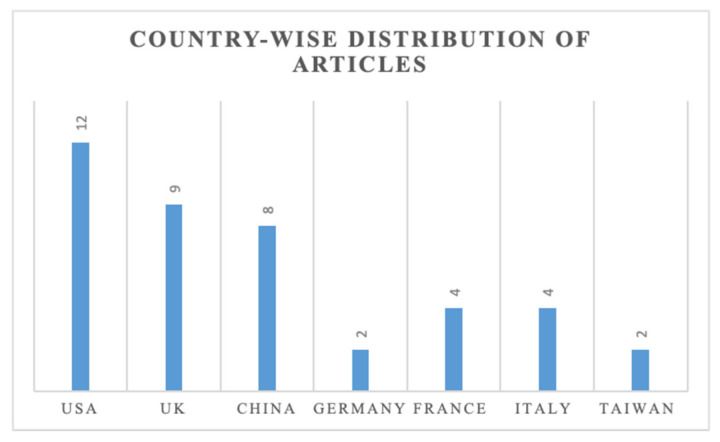
Country-wise distribution of articles.

Considering the studies’ geographic focus, it could be seen that most of the studies took a global approach. The majority of the studies have been conducted in the USA, followed by the UK and China. Figure 3 shows the country-wise distribution of the articles.

### 3.3. Journal Ranking Distribution

Moreover, taking the type of journals based on ABS ranking 2018, it can be seen in Table 3 that journals with ranking 3 were higher (n = 14), whereas journals with ranking 4 accounted for a total of 3. The rankings 3, 4, and 2 indicate that these articles are well researched and heavily referred to. Besides, the selection of 1 ranking indicates that the selection is not biased to the top ranking, and all levels of papers are selected to make it comprehensive. Table 3 represents the number of articles selected from each journal for the analysis.

### 3.4. Topic Analysis

**Figure 4:**
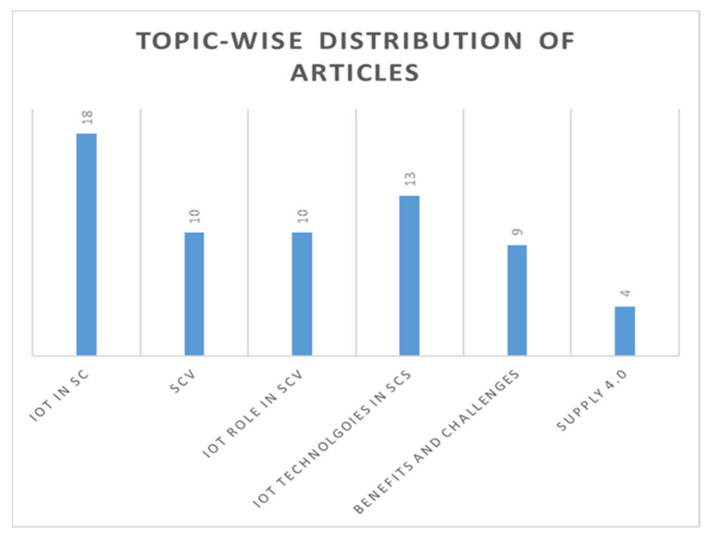
Topic-wise distribution of the articles.

The publications gathered were further categorised based on the topics most discussed within the articles. Figure 4 shows the topic-wise distribution of the articles. It is worthy to note papers discussed multiple issues, as depicted in the figure. IoT in SCs has been given more attention by researchers, followed by the key IoT technologies adopted. Supply 4.0 has been discussed in fewer publications, which does not necessarily mean the topic is less important but may indicate the criticality of the concentrated issues.

### 3.5. Research Method Analysis

**Figure 5:**
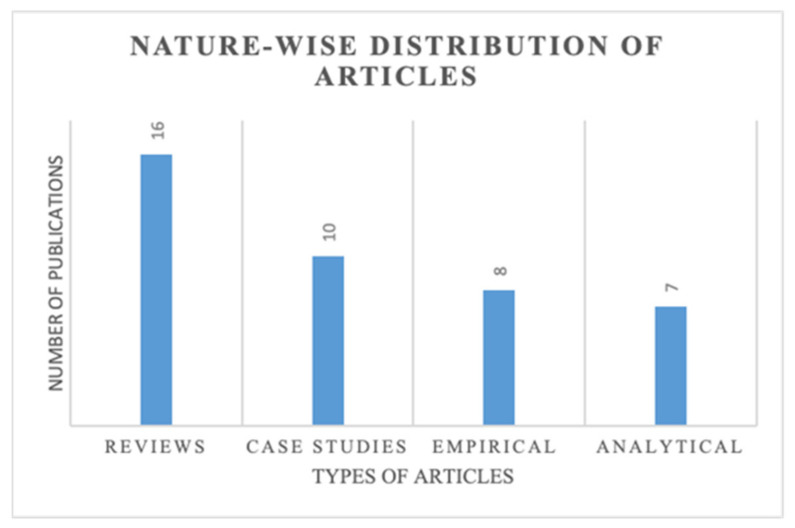
Nature-wise distribution of articles.

**Figure 6:**
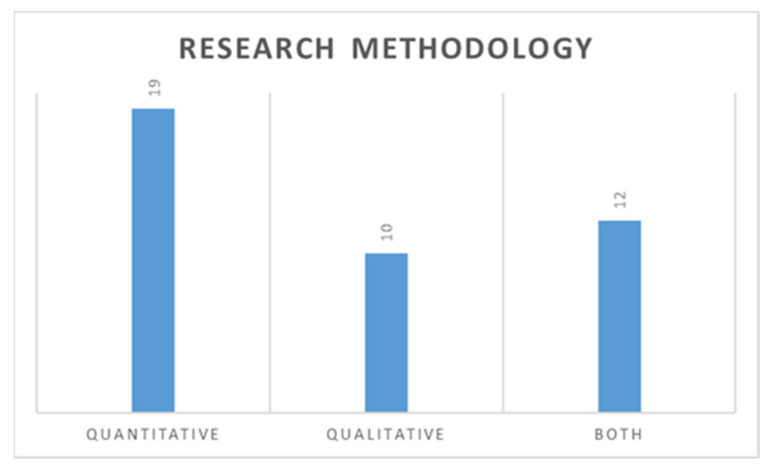
Distribution of adopted methodology.

Figure 5 shows the distribution of selected articles based on the methodological approach used by the researchers. To effectively understand and implement IoT and SCV within Supply 4.0, there is a need for more quantitative, evidence-based analysis at all levels. It is evident from Figure 5 that 16 studies adopted reviews, followed by ten case studies, empirical encompassed eight, while analytical consisted of seven studies. Figure 6 shows the methodological distribution of the articles under study. It is revealed that 19 papers adopted a quantitative approach, 10 used qualitative, and 12 used both quantitative and qualitative methodology.

### 3.6. Word Clouds

**Figure 7:**
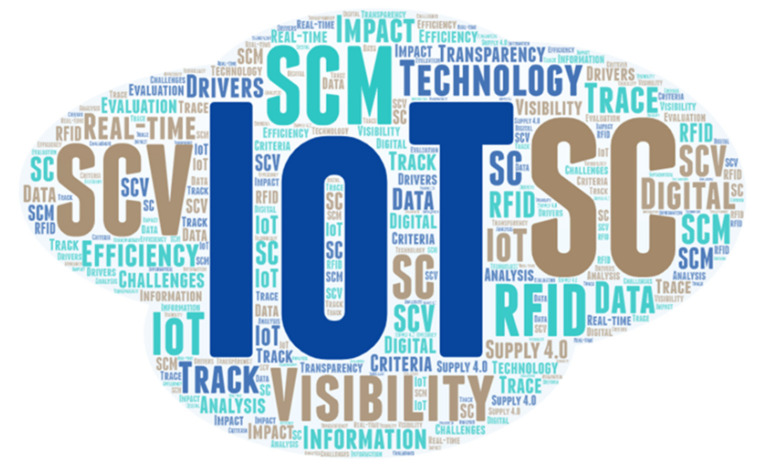
Word cloud showing key themes within the articles.

**Figure 8:**
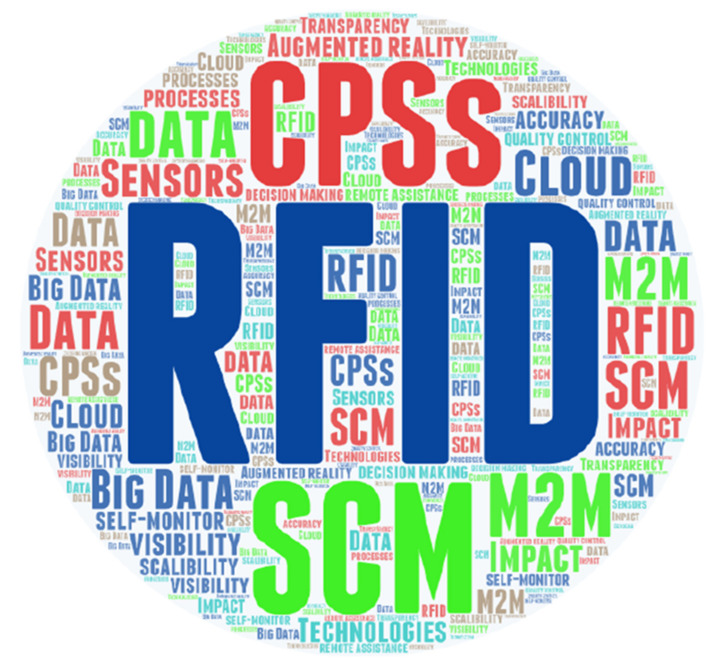
Word cloud showing key recurring themes of IoT technologies within the articles.

Figure 7 presents the main themes in the literature review regarding the rate of occurrence of the terms. These terms include IoT, supply chains, SCV, SCM, visibility, RFID, efficiency, real-time information, digital, technology, and data analysis, which have shown high recurring themes. This indicates that there is a link between IoT, SCV, and efficiency within supply chains. In addition, it was observed that the terms impact, track, and trace are prominent within the data, showing the importance of IoT technologies on SCV in terms of track and trace abilities of these technologies. Figure 8 depicts the key IoT technologies mentioned within the articles according to the rate of occurrence. The terms RFID, CPSs, M2M, SCM, Big Data, augmented reality, and sensors seem to be prominent within the data, which shows that these are the key technologies used in SCs. The occurrence of decision-making, visibility, and accuracy indicate the importance and benefits of using these technologies in SCs.

## 4. Related Literature Reviews

Several systematic reviews have been conducted on the topic of IoT and SCs. This section summarises the most relevant studies and explains the difference between our review and these studies. The study of [39] focused only on IoT’s engineering perspective in SCs. Haddud et al. [12] surveyed the impact of IoT on supplier integration in SC. Ardito et al. [15] focused on Industry 4.0 from the marketing integration perspective, with no particular attention to more extensive applications beyond the marketing viewpoint. Ali et al. [15] and Aryal et al. [16] paid attention to the digital SCs with advantages and disadvantages from theoretical and industrial perspectives. However, the impact of IoT on the SCV context is missing in this study. Ben-Daya et al. [1] took a detailed look at IoT technologies, directing their research on supply chain impact in various sectors and application areas, with no mention of the impact on SCV (Table 4). It appears that either a fragment of IoT has been the centre of focus in these reviews or they address a specific application area. Our review takes a detailed view on the impact of IoT on SCV.

**Figure 9:**
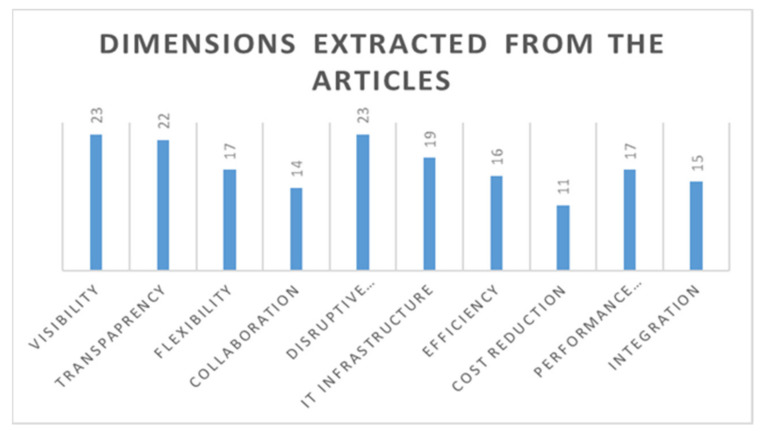
Dimensions extracted from the articles according to the number of occurrences.

Table 5 shows that numerous research studies have focused on the IoT and SCV relationship, and also shows the number of studies focusing on the implementation of IoT in a Supply 4.0 perspective. Several dimensions focusing on IoT in SC have been extracted from these studies, showing that previous research has focused on either one or a combination of these dimensions, but not a single study explores the connection among IoT, Supply 4.0, and SCV. This publication bridges the gap by reviewing the currently available literature and providing guidelines for future research, so the idea of this research remains novel. Figure 9 shows the number of times each dimension has occurred in the articles. This figure shows that most of the articles under investigation focused on visibility and disruptive technology, followed by transparency, IT infrastructure, flexibility, performance measurement, and integration. It was found that collaboration and cost reduction are the dimensions least mentioned within the articles.

### Qualitative Analysis

**Figure 10:**
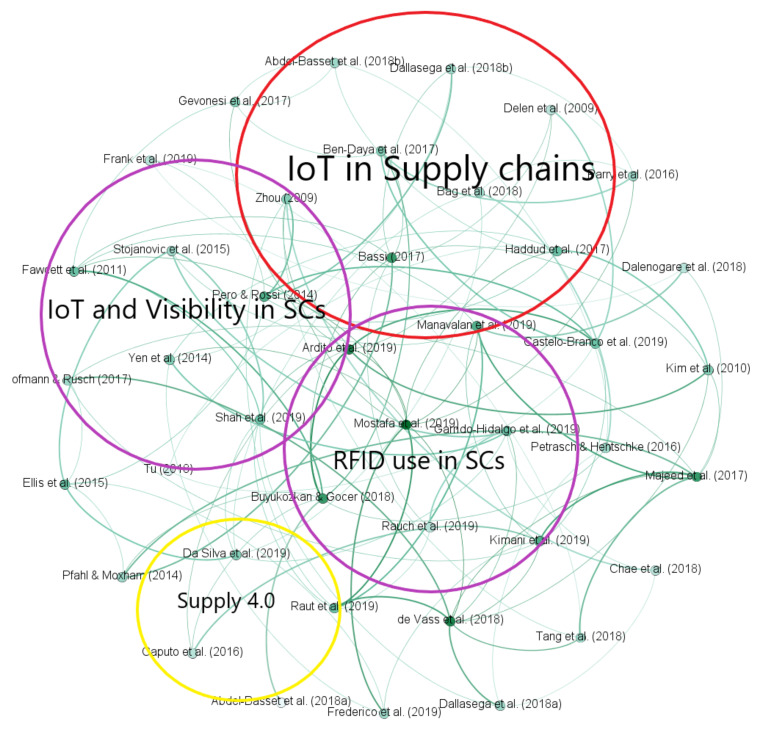
Co-citation analysis using Gephi.

The systematic approach utilised to capture concepts and themes from the selected journals is mentioned as content analysis [34]. This process’s primary emphasis is to identify the relationship between the different variables for generating reliable insights. Hence, the use of content analysis enabled the researcher to pursue an objective and systematic review. The software applied for performing the content analysis was Gephi [1]. Gephi is an open-source visualisation and exploration platform for researchers, where connections between different nodes are visualised and explained. The main advantage of the software is that it allows an in-depth analysis. Figure 10 shows the co-citation analysis of the articles.

Using Gephi, a co-citation analysis of the reviewed articles was conducted to obtain detailed insight on different topics in IoT and SCV and their relationships with each other. Figure 5 shows the graph with the results of the co-citation analysis. Table 4 summarises the top five studies related to these clusters.

Figure 10 shows that there are about four distinct groups of authors that tend to co-occur next to each other. Figure 10 represents four clusters: Clusters 1, 2, 3, and 4 account for 49, 18, 13, and 10% of the papers, respectively. Cluster 1 shows initial research that looks at the use of IoT in SCs. Cluster 2 contains articles that propose IoT benefits in achieving SCV. Cluster 3 groups studies that address radio-frequency identification (RFID) tags used in SCs, focusing on visibility across SCs. Finally, Cluster 4 contains studies that define and explain the adoption of IoT in Supply 4.0. From Figure 5, we can see that the articles in Cluster 4 are independent. In addition, there is a structural gap between Clusters 1, 2, and 3 and Cluster 4, which shows a scarcity of articles discussing the use of IoT in Supply 4.0 to gain visibility. This study intends to fill this gap.

## 5. Discussion

Three significant areas could be found from the content analysis results, and these include the use of IoT, supplier integration, and strategic value. The following section offers a discussion of these areas in detail.

### 5.1. Supply 4.0

Supply 4.0 was introduced to highlight the relationship between Industry 4.0 and the supply chain [5]. It signifies the facilitation of Industry 4.0 to explore and clarify the applicability and the impact it has on supply chains. According to Aryal et al. [8], Supply 4.0 can transform traditional supply chains disruptively; therefore, there are excellent prospects for academic research and contributions in this domain. To add more details, the technologies most associated with Industry 4.0 in the context of supply chains are Big Data analytics, robotics, cloud computing, cyber-physical systems, augmented reality, RFID, M2M, and sensor technologies [5,8,28]. Table 6 gives details about these technologies. These technologies can provide implications to a range of business areas such as new product development, operations, organisational management, business models, etc., which lead to significant changes in the supply chains [12,65]. Swanson [12] states that Supply 4.0 can transform supply chains to create a competitive advantage in terms of product availability, cost reduction, and increase in market share.

Although research in Supply 4.0 is still in its infancy, scholars believe that Supply 4.0 will create considerable benefits to manufacturers [5,8,12,28]. However, it is also argued that to successfully implement Supply 4.0, it is imperative to thoroughly understand the evolution of traditional SCs to Supply 4.0 [66]. Frederico et al. [5] argued that implementing Supply 4.0 requires two key enablers: capabilities and environment. Capabilities regarding digitisation need to be built in the organisation, typically by recruiting specialists. The second key enabler is implementing a two-speed architecture, which means that while the IT mindscape is being modified within the organisation, an environment centred on innovation with a start-up culture is created. A high degree of flexibility and organisational freedom is required to enable rapid development, testing, and solutions. These enablers will provide organisations with fast, flexible, and efficient Supply 4.0.

**Figure 11:**
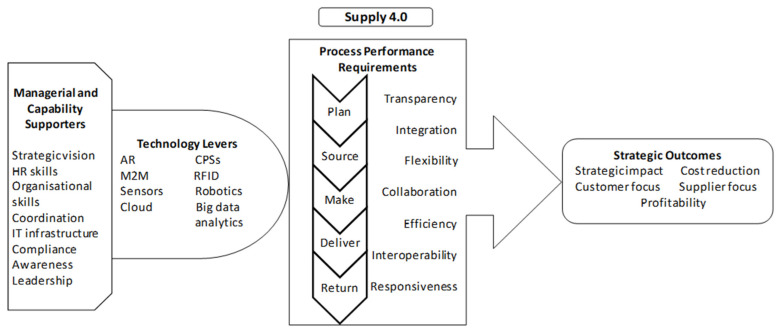
Theoretical framework for Supply 4.0 concept adapted from Frederico et al. [5].

A theoretical framework for the Supply 4.0 concept has been developed by [5], which comprises four layers: managerial and capability supporters, technology levers, process performance requirements, and strategic outcomes (Figure 11). It is believed that organisational and capability supporters help provide support for the development, implementation, and maintenance of Supply 4.0 technologies. The second layer (technology levers) provides support to facilitate the processes to achieve expected performance levels (third layer) to achieve strategic outcomes (fourth layer). Although this framework is supported by some researchers [8,28], further research in this domain is required to clear the cause-and-effect relationship between these four layers in a bottom-up manner. Because of the importance of this framework, there is a need to clarify each of these constructs further.

### 5.2. Use of IoT in Supply 4.0

The introduction of IoT within manufacturing and production environments significantly influences the entire SC [39]. Implementation and integration of modern technology into SCs focuses on essential functions such as procurement, logistics and transport, warehousing, and order fulfilment [5]. In an increasingly digitalised world, IoT devices with mobile capabilities aim to change the SC by providing enhanced revenue opportunities and operational efficiencies [6].

The main aim of IoT technology integrated into SCs is to solve the challenges within logistical operations and synchronise and monitor real-time data from physical processes to cyberspace [50,55]. Different SC functions such as purchasing, transporting, storage, distribution, sales, and returns can be monitored by IoT [27]. IoT devices play a critical role in enhancing relations with vendors through communications in real time [14,60]. IoT allows improved productivity and better working conditions at each SC stage [12,25]. Hence, IoT usage enhances revenue and reduces excess product with less value, enabling quick reaction to changes in client needs or supplier availability and faster deliveries [12,13].

Enhancing product quality, improving equipment efficiency, and helping in the real-time decision-making process can be achieved using data analysis techniques such as Big Data [14]. Specific electronic systems are used to process this collected data [54], stored in the cloud to regulate coordination between different supply chain actors. Supply chains can better target their decision-making processes via this connectivity and easy accessibility of information [10,67]. Enabling commercial activities by IoT focuses not only on the factory environment but also outside the markets [51]. While automation already existed in factories after the third industrial revolution, IoT has enabled greater computerisation, increasing flexibility and efficiency in manufacturing processes [57,58]. It enhances the ability to satisfy customer requirements and increases competitiveness [20].

### 5.3. Key IoT Technologies Used in SC

Key enabling IoT technologies used in SCs include radio-frequency identification (RFID), wireless sensor network (WSN), machine-to-machine communication (M2M), human–machine interaction, etc. [27,47,68]. Increased use of IoT sensors in SCs, such as RFID, makes tracking assets easier, provides more accurate inventories, and enhances a company’s ability to monitor everchanging variables [56]. RFID is an emerging information tracking technology that is highly used in supply chain management [69]. It can disclose information about the product at a low level with an autonomous, instantaneous, and touchless method [7,47]. Unlike barcodes, RFID tags do not need a direct line of vision to transmit data, making it likely to scan different tags as a batch synchronously. Information visibility can be enhanced using RFID to varying SC stages, including acquiring raw materials, manufacturing, logistics, and retail [3,49]. Reduced uncertainty resulting from information visibility and transparency is one of the most potential benefits of RFID. This reduced uncertainty enables vast amounts of benefits such as coordination within SC, reducing inventory, enhancing product availability, and improving the end products’ total quality [70].

Sensors such as RFID are now old technology and have recently received tremendous attention from academics and practitioners. The study conducted by Ellis et al. [9] has shown that information visibility due to regular RFID use has led SCs to achieve a 40–70% reduction in inventory costs alone. Studies have confirmed that SC partners are promised higher SCV by RFID users [1,3,12,64]. However, RFID’s benefits and countless business opportunities are available to SCs that use data information innovatively. The key to gain business value from using RFID relies on the data that is understood and used for decision-making [3]. This viewpoint is found to be prominent in most of the articles. The findings validate that a current problem exists within SCs related to false reads and incomplete information coverage, leading to poor decision-making, which researchers need to address.

It has been found that IoT-enabled analytics is an area of particular interest within SCs, where adoption of IoT has been slower compared to other areas of the manufacturing industry, creating a tremendous amount of opportunity [15,27,58,62,64]. The dynamic nature of SCs and an incredibly competitive environment push all actors in the SC to seek opportunities to enhance performance and create a competitive advantage. Moreover, the required coordination between all actors in the SC can be improved by IoT-enabled analytics, which improves performance and reduces the time taken to gather, scrutinise, communicate, and act upon real-time information [3]. Table 6 highlights some of the key technologies of IoT used in the SC.

### 5.4. Visibility in SC

Visibility across the supply chain has been a hot topic among academics and practitioners. According to Parry et al. [58], SCV is considered from the perception, type, and usefulness of information exchanged or firms’ ability to act on the exchanged information. Transparency and information visibility along the SC can be achieved by adopting IoT [12]. This helps gain accurate real-time information about operations and transactions and frontward and reverse transport of physical objects [12,33].

Supplier integration within SCs is found to be gaining more relevance [72]. The main argument related to the role of supplier integration as presented by these studies is that the development of external and internal linkages enables the creation of a seamlessly connected SC, affecting the competence of the firm in the industry [50,58,70,73]. These connections are aided by IoT technologies such as RFID, where connecting devices through the internet offers a strengthened supplier integration [1,53]. These connections enhance capabilities to recognise, anticipate network, and process information to connect with other devices and services over the internet [45].

Another major prerequisite for pursuing visibility is the coupling of critical technologies and capabilities, both human and technical. Brusset [10] has considered both upstream and downstream linkages for identifying the implications of SC performance. The results indicated the accomplishment of visibility, specifically in the downstream activities, by streamlining the SC activities. Due to the ability to facilitate internal integration with suppliers, IoT has a substantial influence on SCs’ nature and structure by improving communication and collecting and transferring data [1,21]. This helps in effective decision-making and enhances supply chain performance. It also helps manage remote SC operations, better integration with partners for information of the products, and provides more precise information for effective decision-making [8,12]. Besides, the utilisation of internal linkages for extending the downstream visibility could also be noted. Another primary inference is that the firms that exercised supplier integration through IoT were experiencing benefits in the form of higher degrees of flexibility and responsiveness. IoT allows a reduced time lapse between data collection and decision-making that enables SCs to respond to variations in real time, enabling high levels of agility and responsiveness never experienced before [9].

The analysis also led to identifying the influence of information flow in moderating the SCV. The level of sophistication associated with the development of internal linkages affects the information flow [72]. This is because the internal linkages’ efficacy is critical in increasing the decision-making capabilities and supply chain operations using IoT connections [58].

Most of the studies have looked at the role of relational integration in enhancing supply chain visibility. Relational integration is the concept that upholds the personification of the strategic initiatives taken to establish a closer relationship with the limited and selected volume of the suppliers via information collected by IoT devices [10,30]. The other critical facets that should be additionally considered during integration include information, process, and team integration. The underpinning of these elements in the integration process leads to establishing a vital conduit that helps accomplish a high SC performance [72]. This viewpoint is found to be prominent in most of the articles. The findings substantiate that a firm’s IoT capabilities in integrating the suppliers help embed visibility across SCs. Research has also revealed that most of the extant literature exists from the viewpoint of technology. Hardly any study focuses on the link between IoT adoption for enhanced SC performance. Moreover, it could also be understood that SC integration should be regarded as a continuous process. The fundamental connection between IoT adoption and supplier integration is mainly unmapped in the extant literature.

### 5.5. Benefits of Using IoT to Gain SCV

The advent of IoT has led to increased information sharing within SCs through RFIDs [60]. Information sharing through IoT sensors, specifically RFID, is argued to have potential benefit for both retailers and suppliers, which leads to successful interorganisational visibility [27]. The review has shown that the key benefits of using IoT to gain SCV are:

#### 5.5.1. Supply Chain Planning

This involves balancing supply and demand, predicting future needs, and ensuring adequate supply to meet those needs [8]. It includes a demand plan which translates necessary data and information into numerous execution and distribution processes after gathering it in one place [8,42,57]. IoT allows the integration of sensors (for example, RFID) into tools to observe the real-time SC performance, enabling effective planning to adapt to demand and supply changes [3,48].

#### 5.5.2. Collaboration

Collaboration along the SC is required to reduce uncertainty and maintain a high customer service level with minimum possible cost [7,60]. It has identified that external cooperation complications with internal production and inventory control systems result in disconnection between information flow, leading to low SCV [7]. IoT technologies strengthen retailers’ position through effective internal and external collaboration among the SC actors by providing simplified data collection [27,47].

#### 5.5.3. Traceability

Supply chains benefit from implementing IoT by identifying and tracking the components that make up the final product [7,8]. IoT aids organisations to control inventory effectively and efficiently. This consists of tracking inventory levels at different times and receiving alarms when the stock levels run low [12]. To facilitate effective inventory monitoring, IoT technologies have allowed SCs with the ability of inventory-level dynamic rerouting and rebalancing through a standard universal scan and trace capability across all transition points in SCs [2,14,48,64].

#### 5.5.4. Transparency

Through transparent supply chains, the end users of products become conscious of products and services [62]. The use of IoT in SCs has enabled organisations to bring high levels of transparency, as all the actors in SC will have information about their products and source [2,14]. Transparency is vital to gain accurate real-time information on operations and transaction and the frontward and backward movement of physical entities in the SCs [12,41,44].

#### 5.5.5. Flexibility

Flexibility is the capability to react to fluctuations taking place in the SCs, which are controlled and regulated through available real-time data and information [14]. IoT enables SCs to have flexibility at different production stages based on real-time information about demand and supply [12,44]. In addition, data availability allows the upsurge of the production process by identifying disputes and deficiencies within SC [62]. Moreover, real-time information can help achieve product flexibility to increase product offering and product mix to meet changes in demand and supply [5,47].

#### 5.5.6. Performance Management

IoT capabilities such as control systems, performance indicators, Big Data and data mining techniques, and machine learning have the objective to recognise flaws in the SC processes, thus enabling companies to effectively manage their performance [14,62].

#### 5.5.7. Order Management

Order processing can occur through digital means to reduce costs and improve the customer experience [14,39]. Order management through IoT also leads to enhanced delivery speed of inputs and products among different phases of SCs to meet the clients’ changing demands [8,14].

### 5.6. Challenges in Adopting IoT to Gain SCV

#### 5.6.1. Lack of Standardisation

Developing the ability to communicate with each other is the most common technical challenge occurring in an environment, mainly where a large number of different types of devices and technical profiles operate (e.g., autonomous vehicles and drones), which are produced by thousands of diverse brands (each with their standards) [32,44]. This lack of standardisation of IoT devices may lead to inequality in access to valuable data [14,23].

#### 5.6.2. Security

When networked together to share information on the cloud, vast amounts of smart devices become exposed to security risks [55]. These risks pose threats ranging from personal devices to complex IT systems, making individuals and organisations vulnerable to financial and operational damages [13,49]. Similarly, insufficient data injected into the IoT system can be as damaging as data extracted from the system through a data breach [12,59]. Therefore, both systems and communications need to be secure in SCs.

#### 5.6.3. Misread Information

The main challenge in managing vast amounts of data in this digital era is handling data that reveals the quality and efficiency-related factors rather than collecting different unusable data [12]. SCs need to prepare for data challenges such as plans for availability, privacy, storage management, and practical data mining [20,63].

## 6. Lesson Learned

It has been revealed that the majority of the papers discussed the benefits of IoT in SCs with a specific focus on the use of RFIDs across the value chain. The concept of Supply 4.0 will allow firms to be more accurate, more efficient, and more granular by implementing digital performance measurement systems, automation of both physical tasks and planning, and real-time end-to-end transparency throughout the supply chains. It was found that IoT integrated into SCs solves the challenges related to logistical operations and synchronises and monitors real-time data from physical processes to cyberspace. Although the key enabling IoT technologies used in SCs include a variety such as WSNs, M2M, and human–machine interaction, RFID is an emerging information tracking technology highly used in SCM. IoT plays a critical role in achieving visibility across the SCs, as studies have shown a 40–70% reduction in inventory costs alone due to information visibility gained due to regular use of RFIDs. In addition, it was learned that supply chain planning, collaboration, traceability, flexibility, transparency, performance management, and order management are the key benefits of integrating IoT to achieve SCV. Lack of standardisation, security, and misread information poses a threat to achieving SCV through IoT.

## 7. Conclusions, Limitations and Future Work

By pursuing an SQLR, it was aimed to attain significant perceptions about IoT’s crucial role to gain visibility in supply chains. Though all of the chosen articles have deemed the inevitable purpose of adopting IoT to achieve visibility, it could be seen that the papers which specifically focused on the creation of strategic value were few. The main observation is that there are distinct benefits and challenges about adopting IoT in SCs to gain visibility and the benefits of visibility, along with the SC.

Authors have recognised IoT technology’s usefulness to enhance supplier integration as a critical metric for transcending visibility across the network. However, only a few papers delineated the role of IoT complexity and real-time tracking by these technologies on the transformation of the SCs. These studies do not explicitly present the relationship between these attributes and strategic value created by IoT adoption in SCV. In addition, it has been revealed that information sharing through effectively connected IoT devices is not enough to achieve significant improvement until data is correctly interpreted for informed decision-making. Current literature is found to lack this research realm.

It has also been found that there are several obstacles to IoT implementation in SCs, from both technological and managerial perspectives. As security and lack of standardisation of IoT devices have been the focal point of the disputes faced by SCs, it was found that there is a lack of existing research which addresses how to deal with these disputes effectively.

Thus, overall, the QSLR supported the research investigating the critical metrics related to IoT’s influence on SCV. Moreover, by identifying the research gaps in the IoT and SCV literature prominent with “responsiveness”-, “information sharing”-, and “real-time tracking”-related studies, future research is directed to extend these contexts. The review suggested exploiting these metrics’ potential on a broader scale and using empirically relevant methodologies such as mixed-method research to generalise the link between IoT and SCV.

This research has both theoretical and practical implications. The review has revealed a theoretical framework with a comprehensive view of all the dimensions that should be considered for the successful implementation of Supply 4.0. However, it was found that in-depth research and clarity in the context of cause-and-effect relationships between the constructs are required. In addition, the constructs would serve as a guide to practitioners who aim to gain excellence in Supply 4.0.

It has been revealed that the majority of the existing literature in SCs centres on the technology viewpoint, and there is a scarcity of empirical studies that focus on the links between adopting IoT and SCV. Moreover, it is agreed that as supply chain integration should be regarded as a continuous process within the SCs, the connection between IoT adoption and supplier integration can be explored in detail. Another domain for future research can be related to the study of several barriers to IoT implementation in SCs from both technological and managerial perspectives, as literature in this area is scarce.

## Figures and Tables

**Table 1:** Keywords and search strings used in SLR.

ID	Query	WoS	EI	SD	Taylor & Francis
1	TITLE-ABS-KEY (Internet AND of AND things AND Supply 4.0) AND (LIMIT-TO (LANGUAGE, “English)”)	18	10	3	2
2	TITLE-ABS-KEY (IoT AND supply 4.0) AND (LIMIT-TO (LANGUAGE, “English)”)	29	15	9	3
3	TITLE-ABS-KEY (IoT AND visibility) AND (LIMIT-TO (LANGUAGE, “English)”)	31	19	11	2
4	TITLE-ABS-KEY (IoT AND Industry 4.0) AND (LIMIT-TO (LANGUAGE, “English)”)	22	10	8	5
5	TITLE-ABS-KEY (IoT AND supply chain) AND (LIMIT-TO (LANGUAGE, “English)”)	16	10	3	2
6	TITLE-ABS-KEY (Supply 4.0 AND industry 4.0) AND (LIMIT-TO (LANGUAGE, “English)”)	10	7	6	4
7	TITLE-ABS-KEY (supply AND chain AND industry 4.0) AND (LIMIT-TO (LANGUAGE, “English)”)	40	35	11	9
8	TITLE-ABS-KEY (supply AND chain AND visibility AND IoT) AND (LIMIT-TO (LANGUAGE, “English)”)	34	39	14	7
9	TITLE-ABS-KEY (supply AND chain AND visibility) AND (LIMIT-TO (LANGUAGE, “English)”)	28	17	5	7
10	TITLE-ABS-KEY (visibility AND Supply 4.0) AND (LIMIT-TO (LANGUAGE, “English)”)	6	5	5	0
11	TITLE-ABS-KEY (drivers AND IoT and supply AND chain AND visibility) AND (LIMIT-TO (LANGUAGE, “English)”)	10	10	13	1
12	TITLE-ABS-KEY (opportunities AND IoT and supply AND chain AND visibility) AND (LIMIT-TO (LANGUAGE, “English)”)	10	6	6	3
Total		254	183	94	45

**Table 2:** Inclusion and exclusion criteria.

Inclusion Criteria	Exclusion Criteria
Empirical research papers on the impact of IoT in supply chain visibility. Papers published between 2006–2020.	Not into the ABS ranking. Non-English language and out of period papers. Non-academic papers such as white papers, industry magazine papers, and personal blogs.

**Table 3:** Distribution of articles in journals.

Journal	No. of Articles
*Supply Chain Management: An International Journal*	6
*Computers in Industry*	6
*Sensors*	4
*Production and Operations Management*	4
*Journal of Intelligent Manufacturing*	4
*European Journal of Operational Research*	3
*Production Planning & Control*	2
*Journal of Manufacturing Technology Management*	2
*Journal of Supply Chain Management*	2
*Benchmarking: An International Journal*	2
*International Journal of Production Economics*	2
*Transportation Research Part E: Logistics and Transportation Review*	1
*Journal of Enterprise Information Management*	1
*Industrial Management & Data Systems*	1
*Operations Management Research*	1
*Telematics and Informatics*	1
*The International Journal of Logistics Management*	1
*Future Generation Computer Systems*	1
*International Journal of Production Research*	1
*Computers & Industrial Engineering*	1
*Technology Analysis & Strategic Management*	1

**Table 4:** Relevant literature reviews and gaps.

Author	Title	Constructs Used	Methodology	Findings	Gap
[1]	Internet of things and supply chain management: A literature review.	Categorised existing literature using methodology, industry sector, and significant supply chain processes.	Systematic literature review	The majority of studies have looked at the IoT impact with limitations on investigative models and empirical studies.	No mention of IoT impact on supply chain visibility.
[8]	Digital supply chain: Literature review and a proposed framework for future research.	Existing literature on digital supply chains in detail from literature and industrial point of view.	Literature review	Identified fundamental limitations and prospects in the digital supply chain, summarised prior research, and identified knowledge gaps.	The impact of IoT on supply chains from a visibility context is missing in this study.
[15]	Towards industry 4.0; digital mapping technologies for supply chain management-marketing integration.	Patent analysis and real-time examples used to categorise technologies most relevant for efficient SCM-M integration.	Literature review	Detailed information on which digital technologies enable SCM-M assimilation in acquiring information, storage, and SCM-M elaboration.	Industry 4.0 has been explored from the marketing integration perspective, focusing on more extensive applications beyond the marketing viewpoint.
[12]	Examining potential benefits and challenges associated with the internet of things integration in supply chains.	Benefits and challenges of IoT integration to the individuals and the entire supply chains.	Survey	Most of the benefits were a contributing factor to SCM’s success, whereas some of the challenges were perceived as crucial hinders to IoT adoption in SCM.	There was no mention of visibility as a benefit or challenge of IoT in SCM.
[39]	Industry 4.0 as an enabler of proximity for construction supply chains: a systematic literature review.	Organisational capacity and the resource-based view of the impact of Industry 4.0 and IoT by industries.	Systematic literature review	IoT played a significant role in improving a secure data system for SCM and tracing the flow of goods from the source to the company and the end-user.	This study has focused only on the engineering perspective of IoT in supply chains.

**Table 5:** Authors with dimensions extracted from the articles.

Articles	Dimensions Extracted from the Articles									
	Visibility	Transparency	Flexibility	Collaboration	Disruptive Technology	IT Infrastructure	Efficiency	Cost Reduction	Performance Measurement	Integration
[3]	x			x			x		x	
[40]	x	x		x	x	x			x	
[15]	x	x	x			x		x		x
[41]		x	x	x		x	x		x	x
[42]	x	x		x	x					
[43]	x		x		x	x		x		x
[1]		x	x			x	x		x	
[44]	x		x	x				x	x	
[10]	x	x			x					
[8]			x			x				x
[45]		x		x			x			
[46]	x			x		x	x		x	
[47]	x		x					x		
[34]		x			x				x	x
[20]		x		x	x					
[39]					x					
[39]	x		x					x	x	
[48]		x		x	x					
[9]	x			x			x			x
[27]						x		x		x
[49]		x	x			x			x	
[47]					x					
[50]	x				x				x	x
[51]	x							x	x	
[52]				x	x		x			
[21]		x			x	x				
[53]	x	x				x				x
[54]			x							
[55]		x							x	
[12]				x			x			
[2]			x		x		x			
[56]	x				x			x		x
[13]		x				x	x	x		
[57]	x		x		x					
[32]		x			x		x		x	
[23]						x				
[58]			x		x				x	x
[59]	x	x			x		x			x
[60]	x		x			x				
[61]		x		x		x			x	
[7]					x			x		
[62]		x			x		x			
[16]				x		x		x		
[14]									x	x
[11]		x			x	x				
[63]	x		x				x		x	
[18]	x	x			x		x			x
[33]	x		x			x				
[22]	x	x			x		x			x
[64]	x		x			x				

**Table 6:** Key IoT technologies used in the supply chain, adopted from Kalsoom et al. [71].

Key IoT Technologies	IoT Impact on SCs	Limitations	Source
Cyber-physical systems	Evaluate real-time information sharing. Self-monitor and govern the processes. Foresee actions or need of users. Self-organising production.	Real-time evaluation of data. The necessity of deploying comprehensive and expensive infrastructure in geolocations.	[13,27,62]
Big Data	Using historical data to provide proactive risk alerts. Reduce issues related to product quality and failure. Flexible in combining data from different sources for business intelligence.	Full exploitation of value by data tracking is a challenge.	[14,23,47]
Augment Reality	Management of emergencies. Enhancing maintenance activities by providing remote assistance and guidance. Providing new ways of design and manufacturing process integration.	Social acceptance, addressing privacy and profitability might be a challenge.	[23]
RFID	Inventory shrinkage. Saving processing, scanning, and recording times. Accurate and timely delivery. Inventory accuracy and shelf replenishment.	Strong collaboration and high levels of participation between different actors within the supply chain are required.	[48,64]
M2M	Progressive benefits to shipper, receiver, and customer. Real-time visibility. Quality-controlled logistics.	Concerns relating to flexibility and automated systems, and secure systems pose a challenge.	[1,16,27]
Sensor Technologies	Autonomous decision-making. Visibility, theft reduction. Reduce repair cost and maintenance downtime through better monitoring. Safety and security.	Real-time data analysis of data originating from sensors is a challenge. Deploying sensors in geolocations and ubiquitous process control.	[16,48,64]
Cloud Technologies	Quality control. Real-time visibility. Enhanced security measures. Adjusting to market volatility by making SCs wary of how resources should be used in the event of a collapse. Increased scalability abilities.	Losing control over the data and data safety on the web and service outages situations are some challenges.	[23,41,46]
